# Self-Organized Fractal Structures on Plasma-Exposed Silver Surface

**DOI:** 10.3389/fchem.2021.816811

**Published:** 2021-12-24

**Authors:** Xuefen Kan, Ke Chen, Cheng Yin, Yu Yang, Minglei Shan, Huanhuan Wang, Qingbang Han, Bingyan Chen

**Affiliations:** The Jiangsu Key Laboratory of Power Transmission and Distribution Equipment Technology, Hohai University, Changzhou, China

**Keywords:** planar fractal structure, diffusion-limited aggregation, hot spot, surface modification, Corona discharge plasma

## Abstract

Planar fractal microstructure is observed on the silver film treated by positive corona discharge for the first time. Due to the abundant positive ions driven by the electrical field of positive polarity, surface modification is mainly induced by the plasma oxidation effect, resulting in a large scale of dendritic pattern with self-similarity and hierarchy. In contrast, negative ions dominate the plasma-film interaction under negative corona discharge condition, leading to a different surface morphology without fractal characteristics. A growth model based on the modified diffusion-limited aggregation (DLA) theory is proposed to describe the formation of the dendritic fractal structure, whilst the physics behind is attributed to the electric field directed diffusion of the positive ions around the surface roughness. Numerical simulation verifies the high density of the hot spot in the dendritic pattern, which may enable potential applications in fractal photonic metamaterials.

## Introdution

Micro/nano-structures are of great importance for the ability to promote light–matter interaction, facilitating the rapid development of optoelectronic devices and bringing broad application prospects (Gabrielli et al., 2009; Kuznetsov et al., 2016). Concurrent with the rapid development of structures with regular geometries, there has also been an particular interest and effort to utilize the fractal structures for the unique features such as self-similarity and hierarchy (Tikhomirov et al., 2017; Karaca and Baleanu, 2020; Tang et al., 2020; Zeng et al., 2020). For instance, the gold snowflake-like fractal metasurface is applied in graphene photodetectors with broadband and polarization-insensitive plasmonic enhancement (Fang et al., 2016). A plasmonic leaf with fractal structure can enhance light-matter interaction and is expected to be utilized in a variety of applications (Liu et al., 2019a). Miniaturized fractal optical nanoantennas have been proposed and can reach a main resonance regime of sub-micron wavelength (Seitl et al., 2020). Fractal plasmonic black gold prepared by the overcurrent electrodeposition method also shows a broad-band absorption (Yu et al., 2020). Particularly, planar fractal metallic structures may exhibit multiband electromagnetic response in a broad frequency range, which has drawn much interest in the design of novel photonic metamaterials.

At present, there exists a wide variety of way to produce fractal structures. For example, Raveendran et al. report that the noble metal nanodendritic substrates are formed by electrodeposition method (Raveendran and Docoslis, 2020; Raveendran et al., 2020; Raveendran and Docoslis, 2021) and demonstrate that silver dendrite fractal nanostructures prepared by a facile electrochemical deposition method (Cheng et al., 2019). Besides, hydrothermal etching method has been used to synthesize dendritic silver nanostructures (Chan et al., 2013). Yang et al. also use a replacement reaction method to produce fractal nanostructures (Yang et al., 2020). But the high-cost of the micro-nano fabrication, the poor stability, and the long synthesis period still hinder the mass production of these fractal structures. There is still an urgent demand for a simple, convenient and cost-efficient method to prepare planar fractal structure with high stability.

In this work, we report the formation of planar dendritic fractal microstructure on a silver surface treated by positive corona discharge at atmospheric pressure. Recently, much effort has been paid to atmospheric plasma for its application in surface treatment and nanocomposite synthesis (Levchenko et al., 2009; Liu et al., 2019b; Chiang et al., 2019; Sun et al., 2020). Especially, plasma has become the most effective tool to promote contaminants degradation in water environment (Guo et al., 2021a; Guo et al., 2021b). However, to the best of our knowledge, no fractal structure has been observed on the treated surface via atmospheric plasma. Our experiment further reveals that by altering the discharge polarity, a completely different surface morphology is formed, where the plasma products of irregular shape scatter on the film surface in a complete random manner. A growth scenario which enables us to explain the main experimental observations is proposed. Under positive corona discharge, positive ions are driven by the localized electrical field and responsible for the surface oxidation, whilst the dendritic growth of the fractal structure can be well described by a modified diffusion-limited aggregation (DLA) model. This work provides a new method to create self-organized fractal structures on plasma-exposed silver surfaces, which may offer opportunities for cost-effective and straightforward nanofabrication of various fractal photonic devices in the future.

## Experiments

**FIGURE 1:**
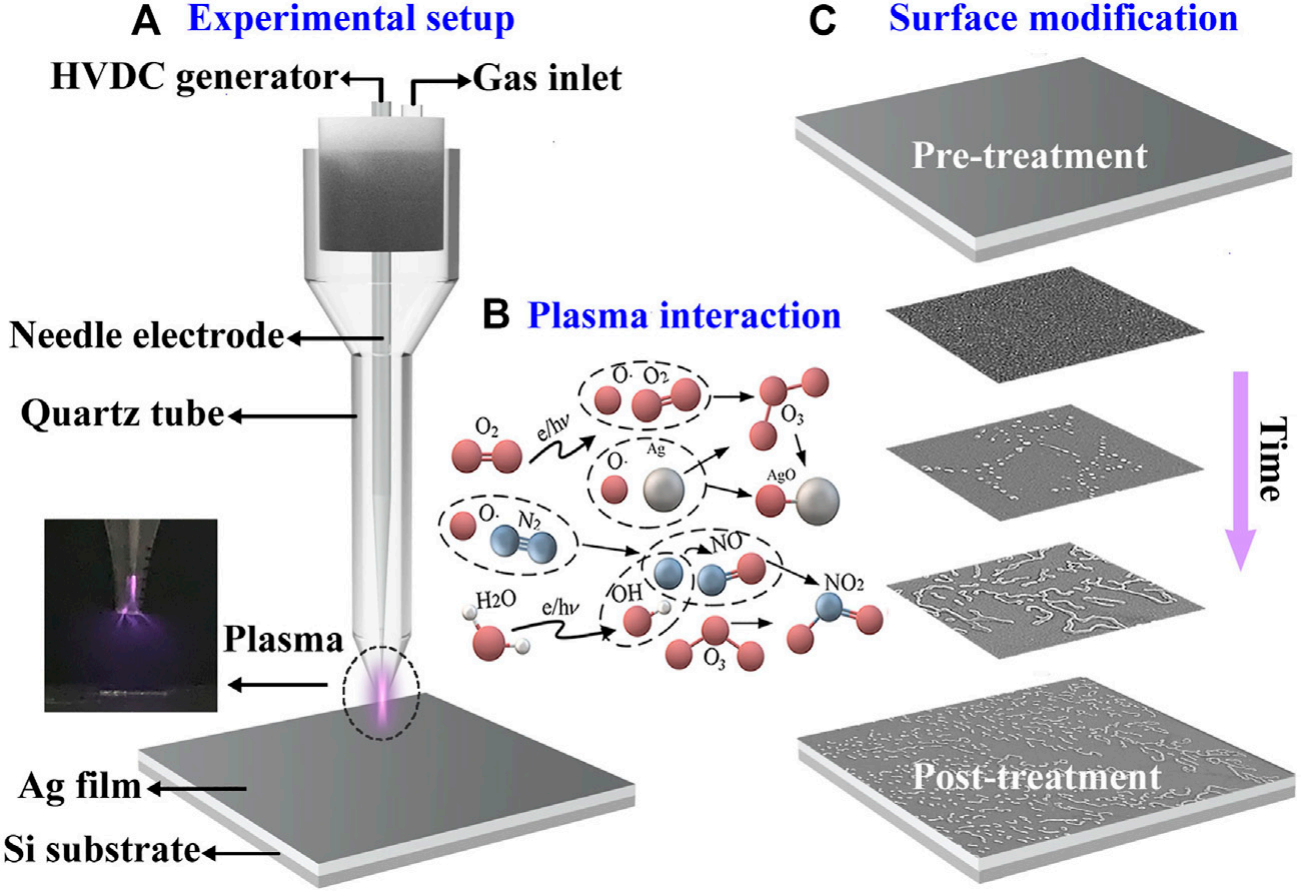
**(A)** Schematic diagram of the experimental setup; **(B)** Related chemical process; **(C)** Procedure of the surface modification of the plasma-exposed silver film.

First, silver films are deposited on the silicon substrates of 1 cm^2^ by magnetron sputtering (JGP-450A, Sky technology development) and the deposition thickness of the film can be precisely controlled. The setup of the microplasma system used for surface modification is illustrated in Figure 1A. A high voltage direct current (HVDC) power source (Teslaman, TCM6000) is applied to ignite the plasma and switch the discharge polarity in an open-air environment. During the experiment, the excitation voltage and current are fixed at 18 kV and 22 μA , respectively. Through the gas-inlet, the air flow at 40 sccm is supplied to the microplasma system. The corona discharge reactor is composed of a stainless-steel needle-like anode of 1.8 mm diameter and a tapered quartz tube for better focusing of the generated plasma. The inset of Figure 1A shows the photo of the plasma and the sample substrate, whilst the latter is linked to the grounded outer electrode. The distance between anode and substrate is approximately 1 cm, which can be adjusted by a two-dimensional translation stage. The chemical reaction between plasma and silver film is shown schematically in Figure 1B, where lots of reactive species are induced by high voltage discharged plasma in free space (Guo et al., 2021a). First, oxygen decomposed by high energy electrons can form oxygen radicals (O) (Jiang et al., 2020), which can further react with oxygen molecules to form ozone (O_3_) (Zhou et al., 2020). Both oxygen radicals and ozone play an important role during the surface oxidation of the silver film, which produces Ag_2_O_2_ (El Mel et al., 2019; Kayed, 2021) in ambient temperature. In addition, hydroxyl radical (OH) and nitrogen oxide (NO_x_) are generated via the excitation and ionization of water and nitrogen in air (Wandell et al., 2019; Brisset et al., 2021). Both products can contribute to the oxidation of silver film through further reaction. Figure 1C shows the step-by-step modification of silver surface under positive corona discharge, which is the key subject of this paper. The scanning electron microscope (SEM, Hitachi, S-3400N) images highlight two interesting observations: (i) In the beginning, separated dots disperse randomly on the silver surface due to the plasma oxidation effect. (ii) Curves of certain length begin to appear via the successive interconnections of several dots, which eventually turn into dendritic-like fractal patterns over the whole plane.

## Results and Discussion

**FIGURE 2:**
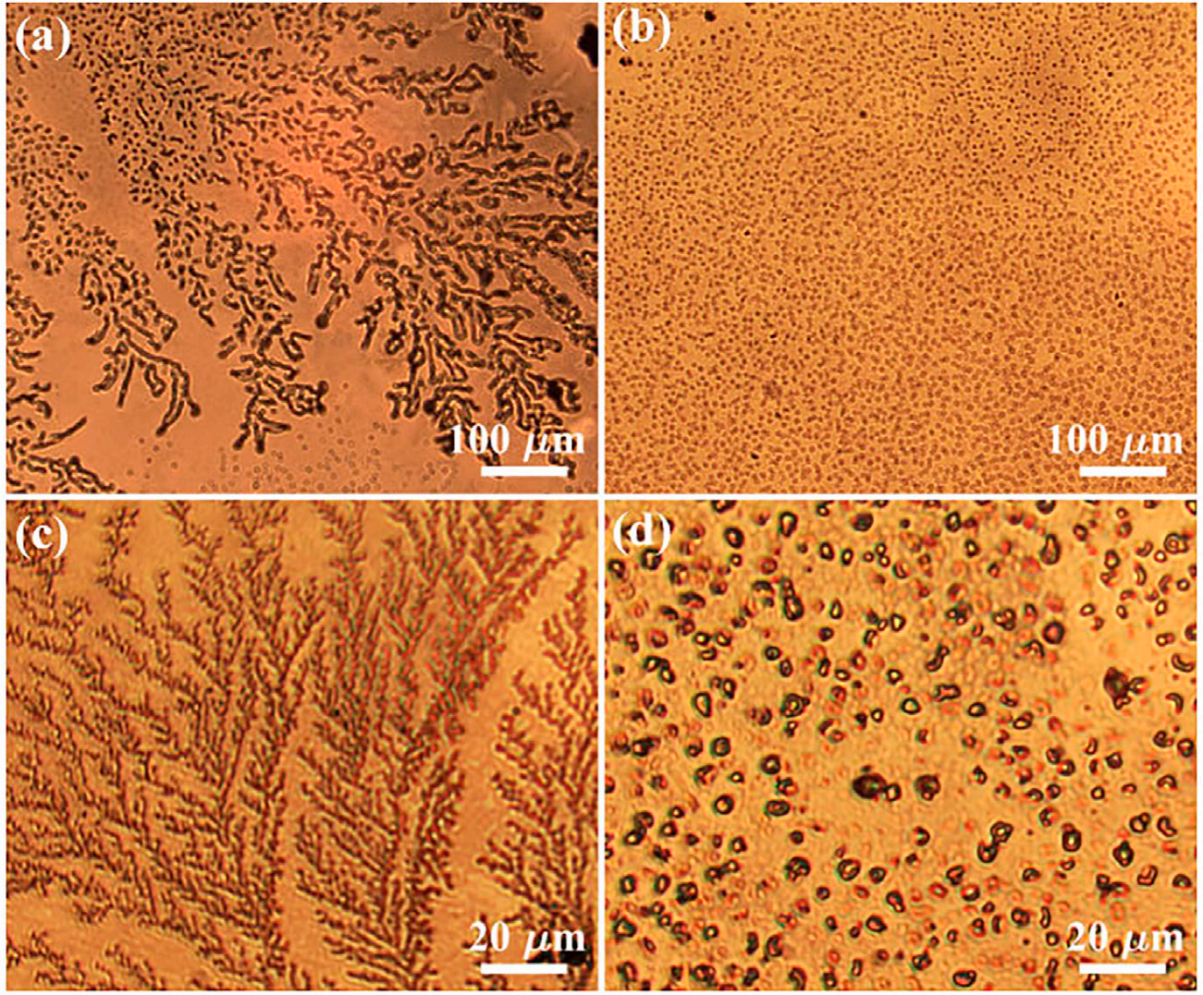
The microscopic images of the surface morphology of silver films after plasma treatment. **(A)** and **(C)** are typical fractal structures using positive corona discharge; **(B)** and **(D)** are typical random structures under negative corona discharge.

The microscopic images of typical morphologies on the silver films exposed to corona discharge plasma under different discharge polarities are compared in Figure 2. As can be seen in Figure 2A, branches extend outward along the main stem, and the overall structure under positive corona discharge appears as a complex and dendritic pattern. A rather similar pattern can be observed in Figure 2C under 5x magnification. The obvious self-similarity and hierarchy are the most basic and important characteristics of fractal objects. Although surface modification via plasma such as plasma etching has been already widely investigated and applied, no fractal structure is reported before to the best of our knowledge. For comparison, the random structures on the silver surface exposed to negative corona discharge are shown in Figures 2B,D at different magnification. Dot-like products of irregular shape scatter evenly over the surface, and few linear or branched connections can be found. By switching the discharge polarity, the surface morphologies under positive/negative polarity are completely different from each other.

**FIGURE 3:**
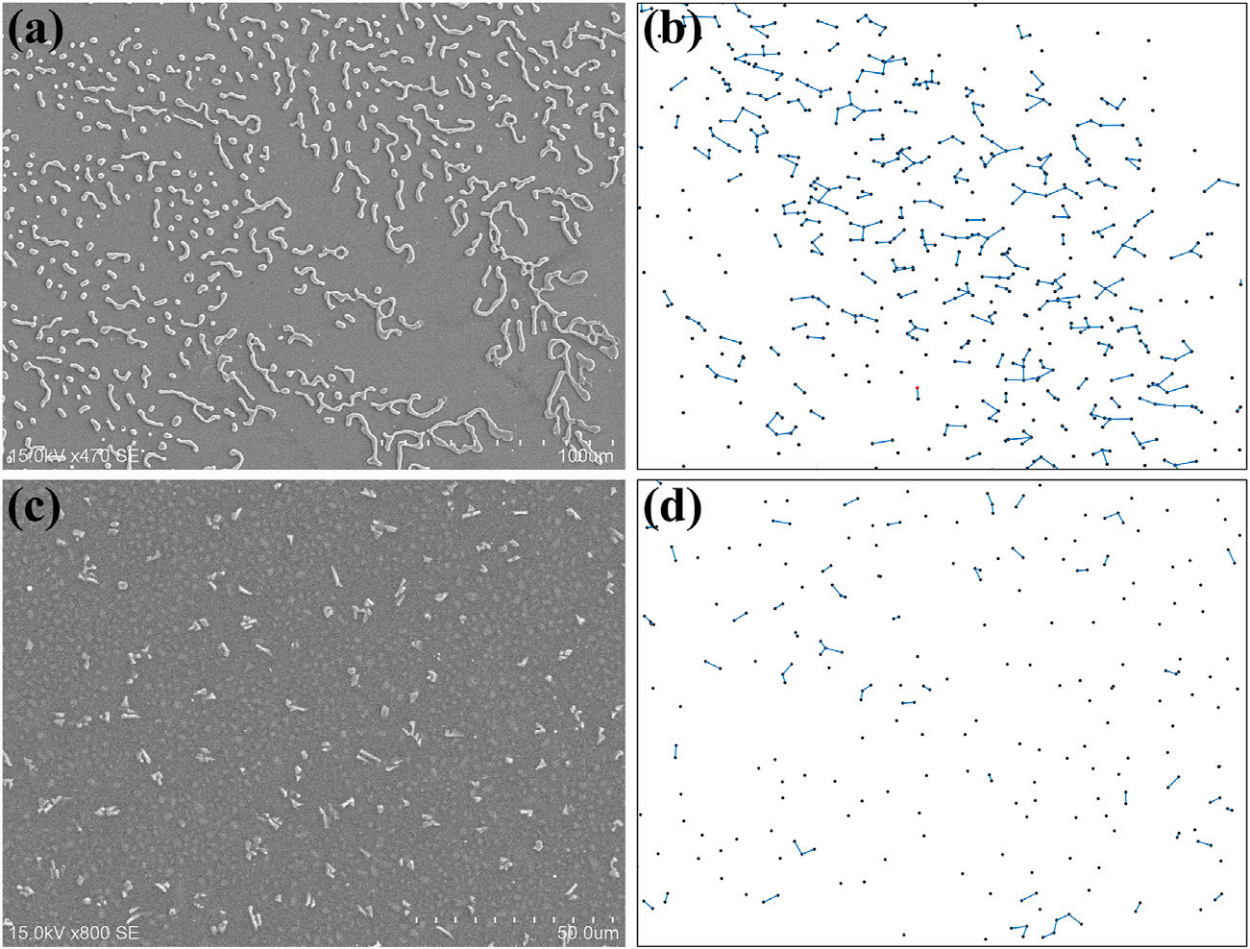
**(A)** The SEM image of the surface pattern and **(B)** the simulated growth scenario of fractal structure under positive corona discharge; **(C)** The SEM images of the surface pattern and **(D)** the simulated growth scenario of random structure under negative corona discharge.

At this stage, a simple growth scenario of the surface pattern based on a modified DLA model is proposed. Figures 3A,C show the SEM images of different surface morphologies on the plasma-exposed silver films under positive/negative corona discharge, respectively. For positive corona discharge, the dendritic-like fractal pattern is composed of various curved lines of different length. According to Figure 1C, these curves do not take the shape as a whole, instead their length increase gradually as more scattered dots are connected. Chemically, all the substance forming the fractal structure are product of the plasma induced surface oxidation. Hence, it is natural to deduce that two very close dots would be connected to create a curve when oxidation reaction takes place. Since the dot-like and curve-like structures extend above the substrate surface level, further oxidation reaction inclines to occur in the immediate vicinity of these irregularities.

Based on random diffusion and irreversible binding, the DLA theory (Witten and Sander, 1983) has been successfully applied to the formation of various branched fractal structures, such as crystallization, evaporation induced aggregation, and so on. In our case, the DLA model is modified by replacing the binding condition with the attraction effect among the particles. During the simulation, hundreds of particles appear on the screen one by one, and a line would be added if the distance between two particles is less than a threshold. These particles and lines represent the surface irregularities due to plasma oxidation under positive corona discharge. The key idea of the modified DLA model is that the dropping position of a particle is not completely random. Since the attraction effect exists between these particles, the latter dropped particle has a higher probability to be found in place close to an existing particle. In our model, an exponential decay dependence of the probability over the distance from any existing particle is applied. Supposing that the position of $i_{th}$ particle is denoted by $r_{i}$ , then the relative probability P of finding this particle at a specific position r is given by

$$P\left(r\right)=\frac{1}{\beta }\sum_{n=1}^{i-1}e^{-1/\beta \left|r-r_{n}\right|}.$$ (1)

where β denotes the average distance between two adjacent particles, and its value is set as 10μm in the simulation. The numerical result shown in Figure 3B is very analogous to the SEM image, which confirms that surface oxidation occurs more frequently near these surface irregularities. By comparison, no obvious attraction exists between the irregularities under negative corona discharge condition, resulting a random distribution on the whole surface. The corresponding simulation result shown in Figure 3D resembles with the related SEM image in Figure 3C.

Obviously, the attraction effect between the particles is crucial to the formation of the fractal pattern in the numerical simulation of Figure 3B. The physics behind can be attributed to the electrical field directed diffusion of the plasma products towards the film surface. Since the plasma induced film roughness is above the surface level, a two-dimensional electric field along the surface is created correspondingly in the plasma area. The strength of the localized electric field is related to the curvature of the microstructure, which can be extremely higher at small radius. On the other hand, high voltage corona discharge produces various reactive species, such as electrons, ions, and free radicals. But electrons have little effect on the surface modification since they are simply transported through the grounded substrate. The chemical function of the negative ions is reduction, so only positive ions with strong oxidation effect will be discussed in the rest of the paper.

**FIGURE 4:**
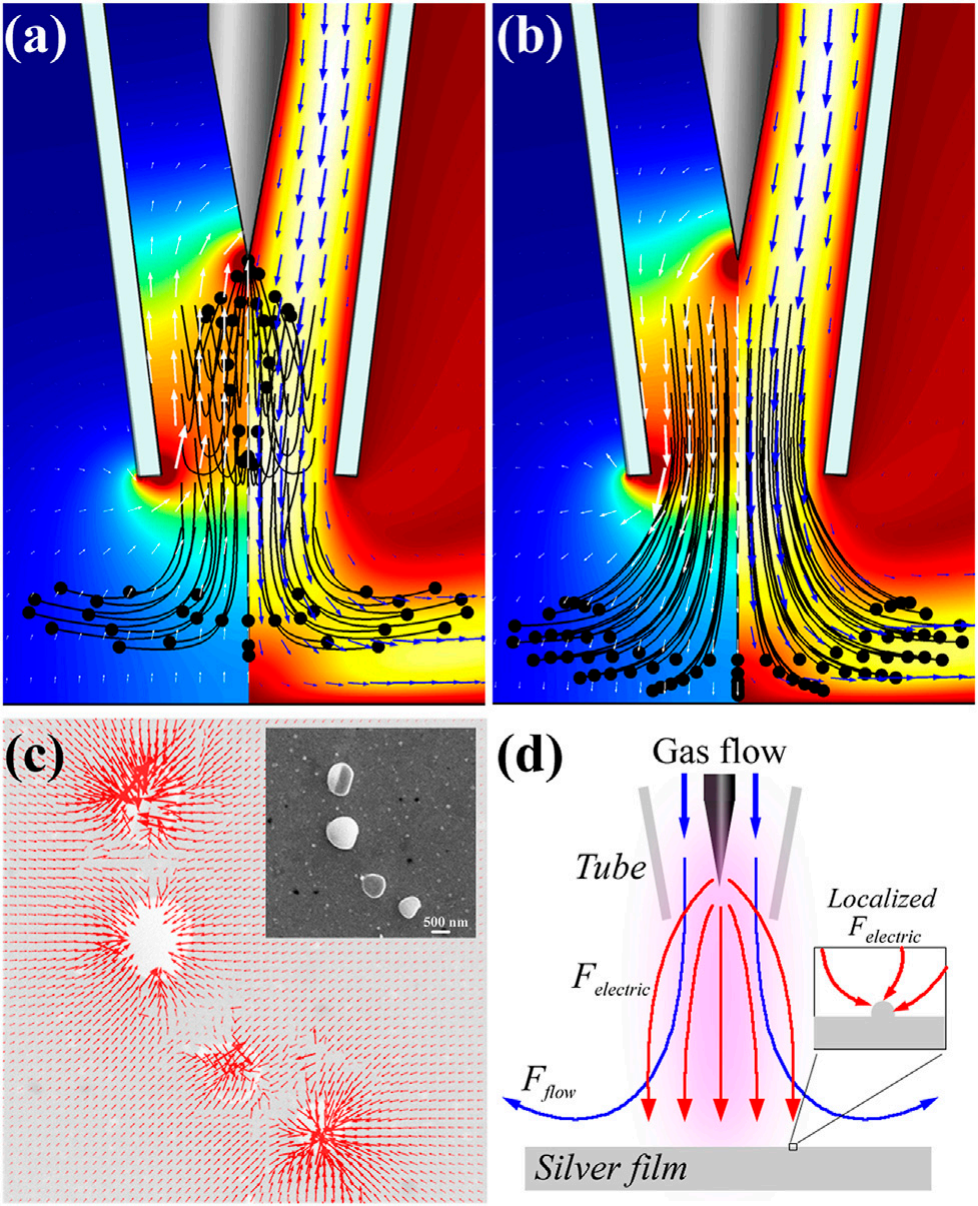
The electrical field directed diffusion of the plasma products between the needle-plate electrode and silver film. **(A,B)** The electrical potential **(left)** and flow velocity **(right)** distribution above the silver film. White and blue arrows denote the directions of the electrical field and the gas flow, and the black lines and dots represent the trajectories of positive ions. **(A)** and **(B)** correspond to the negative/positive corona discharge, respectively; **(C)** Simulated two-dimensional profile of the microscopic electrical filed at the film surface with the same nanoparticles distribution as shown in the inset; **(D)** The schematic of air flow (blue line) and electric force (red line) acting on positive ions in the plasma region (purple shadow), and the inset shows that the microscopic electric field is directed to surface irregularity.

Combined with the DLA model, a growth mechanism of the fractal structure is established to explain the main experimental observations. In the plasma system, the major force exerting on the positive ions is the electrostatic force $F_{electric}$ , whilst the gas jet ejected from the quartz tube produces an additional force $F_{flow}$ on the positive ions independent of the discharge polarities. Hence the total force exerted on the positive ions is $F_{electric}+F_{flow}$ . For positive corona discharge, the $F_{electric}$ is directed towards the film surface, while negative corona discharge sets the force in the opposite direction towards the needle electrode. In Figure 4, numerical simulations are performed to analyze the trajectories of positive ions under positive/negative corona discharge. The locations where the positive ions hit the surface are of great importance since surface oxidation is more likely to occur in the same place. As shown in Figure 4A, most of the positive ions are drawn to the needle by the electrical field while the air flow blows the rest of ions to the substrate surface. This explains the complete random distribution of the oxidation products under negative corona discharge. To understand the formation of fractal structure under positive corona discharge, the first fact shown in Figure 4B is that all the positive ions are directed to the surface by the combined effect of the electric field and air flow. The second fact can be attributed to the reshaping effect of the micro-electric field. The micro-electric field at the surface is not uniform and invariant during the treatment, instead its inhomogeneity increases continuously due to the increasing surface roughness. As a concrete example, the micro-electric field around four nanoparticles is simulated in Figure 4C with the same pattern as shown in the inset. It is clear that the electric field concentrates to these particles with high intensity, which indicates a strong and localized electrical force $F_{electric}$ in the same direction. Hence the positive ions are attracted to the existing particles by the microscopic force, which induce oxidation reaction to build a connection between adjacent particles. The schematic diagram of the growth mechanism is shown in Figure 4D, where the macro-electric field and the air flow draw the positive ions to the film surface, while the micro-electric field further attract these ions to the surface irregularities.

**FIGURE 5:**
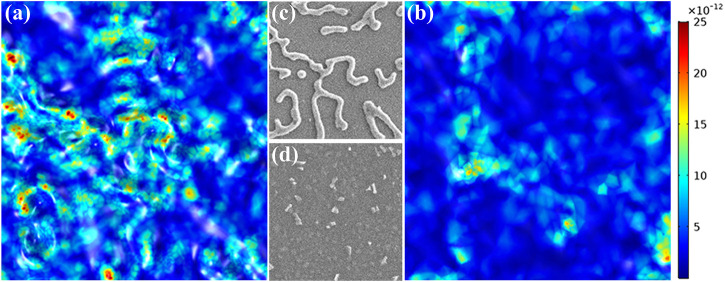
**(A)** and **(B)** are the simulated profiles of the micro-electric field on the film surfaces. **(C)** and **(D)** are the corresponding fragments of the SEM images in the same area. **(C)** and **(D)** are under positive and negative discharge condition, respectively.

The rich electric, magnetic and plasmonic characteristic of photonic materials based on fractal geometry is of particular interest. The proposed fractal microstructure on the silver film significantly increases its surface area to assure sufficient photon capture, which is particularly useful to facilitate various on-chip applications with light-matter interaction. For a concrete illustration, our silver-based dendritic-like fractal structure on a surface can be applied as a highly sensitive surface enhanced Raman scattering (SERS) substrate with low-cost. Using crystal violet as detection molecule, experiments demonstrate an enhancement factor as high as 10^5^ via the positive corona discharged silver surface, which is one order higher than its counterpart by altering the discharge polarity. To discuss the difference under different discharge polarity, we created identical structures for simulation as those shown in the SEM images in Figures 5C,D. And the corresponding electric field at the surfaces are simulated in Figures 5A,B under direct illumination of 785 nm plane wave. Generally speaking, the SERS enhancement depends crucially on the large optical field enhancement due to the hot spots and it is obviously in Figure 5 that the density of the hot spots is much higher on the substrate under positive corona discharge condition. It should note that the intricately connected metallic pattern has an extended bandwidth for surface plasmon resonance due to its fractal-like feature, which exhibits a strong electromagnetic coupling effect. Under negative corona discharge, hot spots are mainly created between adjacent particles, whilst under positive corona discharge hot spots concentrate in the particle junction sites. The hot spot density has been greatly increased by the dendritic-like fractal structure, enabling the plasmon-enhanced light collection from deep ultraviolet to visible light. Besides the hot spots issue, there exists more space between the branches in the dendritic morphology, so the deposition of both small molecules and large analytes such as protein are enhanced. Hence, the plasma exposed silver surface with a dendritic morphology holds a great potential as an economic, effective, and reliable SERS substrate.

## Conclusion

In conclusion, we report the spontaneous formation of fractal microstructure on a silver surface subject to the microplasma. At atmospheric pressure, surface modification via positive corona discharge produces a large-scale dendritic pattern with self-similarity. In contrast, the negative corona discharge generates the completely different morphology of randomly distributed dots. We also propose a growth mechanism to explain how the microstructure morphology can be turned from random scattered dots to fractal dendrites by altering the discharge polarity. Simulation via a modified DLA model reveals the growth process of fractal pattern, and the physics behind is attributed to the electric field directed diffusion of the positive ions with high oxidation activity in the plasma area. This work provides a simple and fast way to produce cost-efficient metallic fractal structure with a high surface area, which may facilitate the on-chip light matter interaction due to its high hot spot density. The fractal nanostructures with unequalled optical performance provide potential applications in low-cost SERS detection and fractal photonic metamaterials.

## Data Availability

The original contributions presented in the study are included in the article/Supplementary Material, further inquiries can be directed to the corresponding authors.
